# Fibromuscular Dysplasia: Three Cases to Highlight a Requirement for Surveillance Strategy Optimization

**DOI:** 10.7759/cureus.50802

**Published:** 2023-12-19

**Authors:** Aaron Tran, Erwin Yii, Anthony E Dear

**Affiliations:** 1 Medicine, Monash University, Melbourne, AUS; 2 Vascular Surgery, Eastern Health, Melbourne, AUS

**Keywords:** surveillance, genetics, vascular disease, artery, fibromuscular disease

## Abstract

Fibromuscular dysplasia (FMD) is a rare vascular disease with broad, potentially severe complications. We present three cases of FMD covering the spectrum of clinical presentations involving the abdominal and visceral vasculature, and highlight the potential role of high-risk genotype detection in assisting with the determination of which patients may benefit from a more aggressive surveillance strategy.

## Introduction

Fibromuscular dysplasia (FMD) is a non-inflammatory, non-atherosclerotic disease predominantly affecting Caucasians and females [1], with sequelae including arterial dissection, occlusion, and aneurysm [2]. With a familial distribution a number of genetic variants are implicated in the pathogenesis of FMD, although the clinical utility of these variants is yet to be definitively evaluated [1,3-7]. We identify three cases of FMD spanning the spectrum of clinical presentation and discuss the potential utility of genotyping FMD patients with visceral artery aneurysms (VAA).

## Case presentation

Case 1

**Figure 1:**
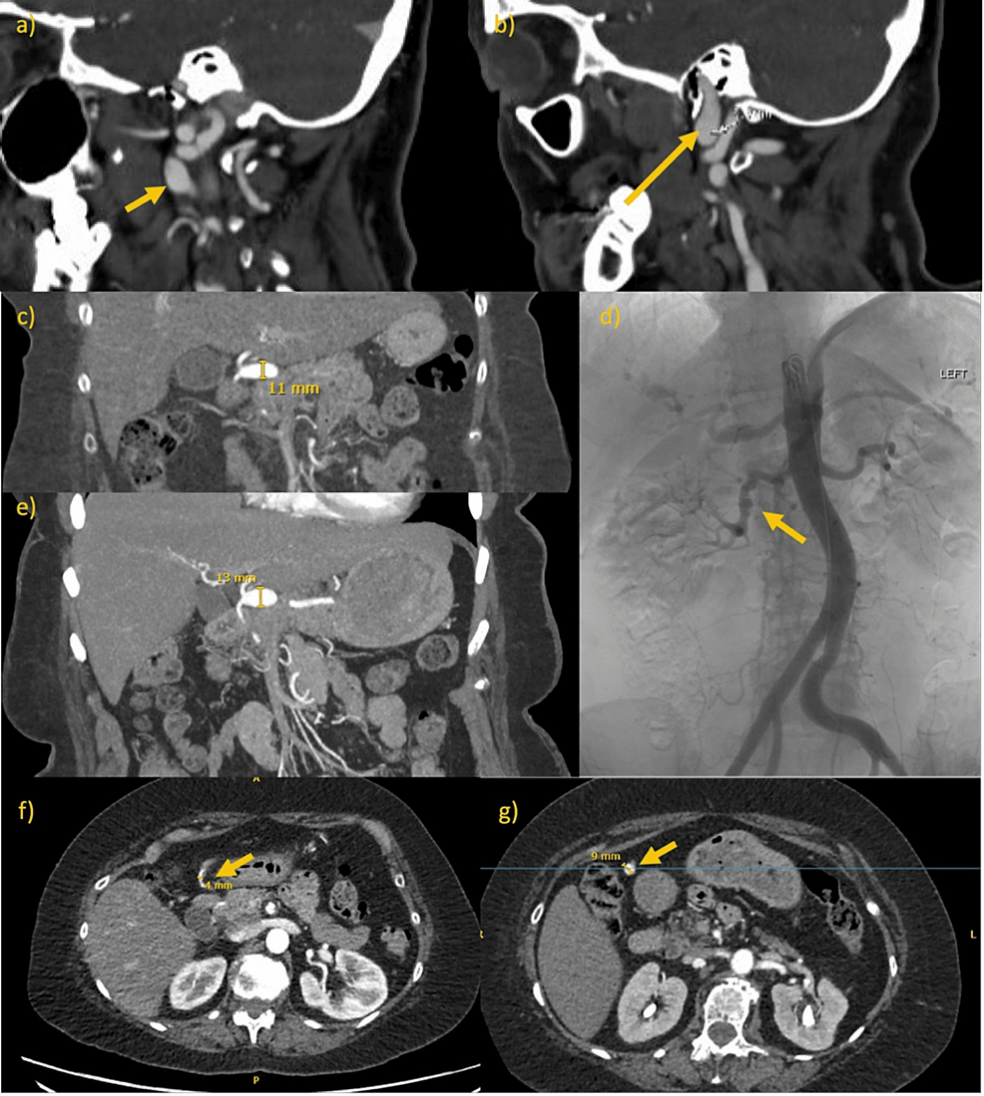
CTA (highlighted by arrows) demonstrating (a) right and (b) left significant distal cervical FMD and fusiform ICA aneurysm (yellow arrows). (c) CTA demonstrating 11mm CHA aneurysm in coronal plane. (d) Abdominal angiogram (highlighted by arrow) demonstrating evidence of FMD with beading of the right renal artery. (e) CTA performed nine months later demonstrating enlarged 13mm CHA aneurysm. CTA (highlighted by arrow) demonstrating GDA in axial plane on (f) presentation and at (g) nine months.

A 59-year-old female with no relevant family history and mild hypertension presented with a one-week history of headache. CT angiogram (CTA) demonstrated bilateral distal cervical FMD, bilateral internal carotid artery (ICA) aneurysms (Figures 1a, 1b), and an 11mm distal common hepatic artery (CHA) aneurysm (Figure 1c). Abdominal digital subtraction angiography (DSA) yielded multifocal beading of the right gastroepiploic and right renal arteries (Figure 1d). Inpatient stay was complicated by non-ST-elevation myocardial infarction (NSTEMI) with angiography demonstrating spontaneous coronary artery dissection (SCAD) of the right coronary artery which was managed medically.

CTA at nine months demonstrated CHA aneurysm enlargement from 11mm to 13mm (Figure 1e), remaining below the threshold for intervention, together with a new 9mm gastroduodenal artery (GDA) aneurysm (Figures 1f, 1g), which was successfully coiled in line with European Society for Vascular Surgery (ESVS) guidelines [8].

Case 2

**Figure 2:**
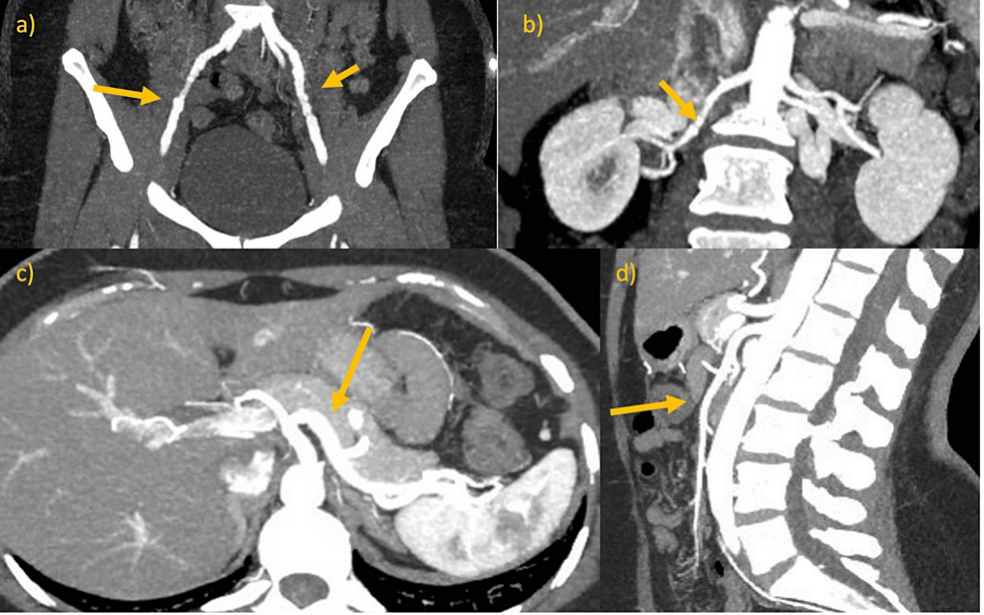
CTA (highlighted by arrows) in coronal plane demonstrating (a) bilateral EIA contour abnormality and (b) mild beading of the right renal artery. (c) Normal coeliac axis in axial plane (highlighted by arrow). (d) Normal superior mesenteric artery in sagittal plane (highlighted by arrow).

A 46-year-old female with no significant family history with a two-day history of central pleuritic chest pain. CTA demonstrated bilateral external iliac artery (EIA) contour abnormality and right renal artery beading, consistent with FMD (Figures 2a, 2b), and no mesenteric visceral artery involvement (Figures 2c, 2d). CTA of the carotids was unremarkable (not shown).

Case 3

**Figure 3:**
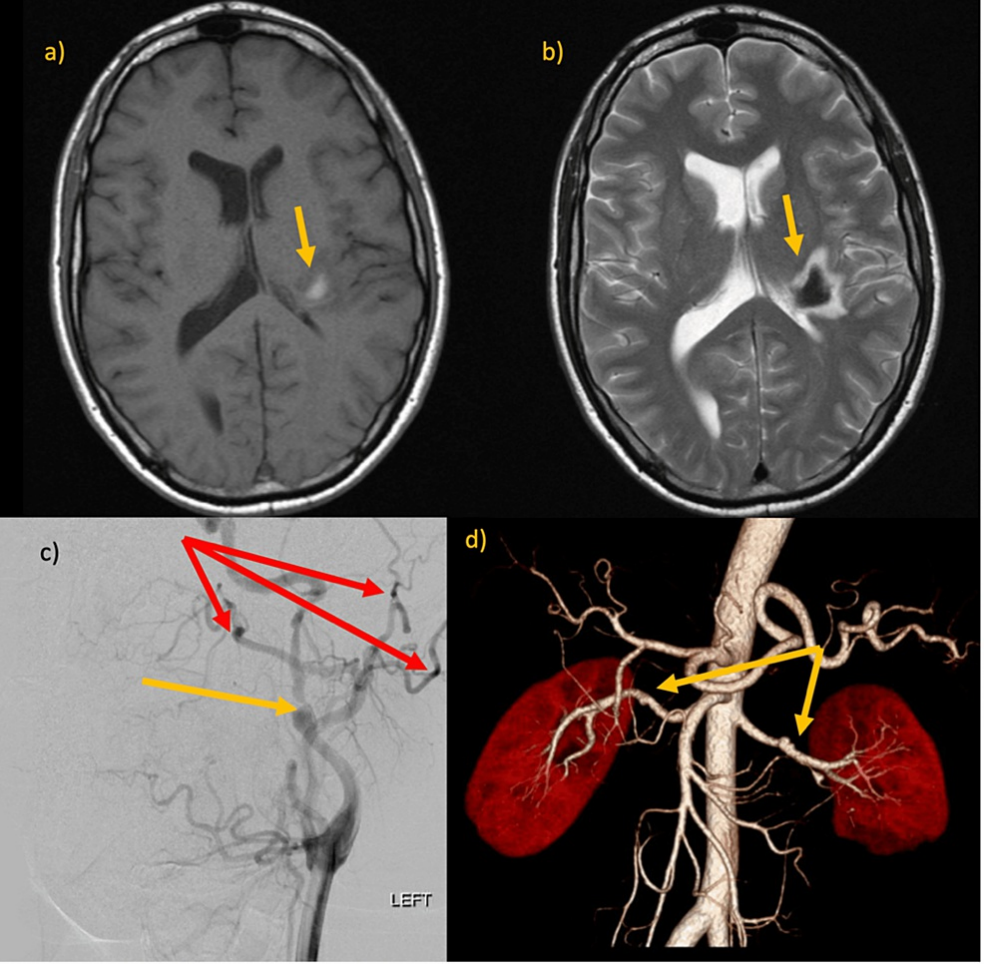
MRI brain T1 (a) and T2 (b) demonstrating left corona radiata acute haemorrhage (highlighted by arrows). (c) DSA demonstrating left ICA high grade stenosis (highlighted by yellow arrow) and left ECA focal irregularities (highlighted by red arrows). (d) 3D reconstruction of CT angiogram demonstrating bilateral renal artery irregularities and normal mesenteric visceral arteries (highlighted by arrows).

A 40-year-old male presented with a three-month history of headache. MRI brain demonstrated acute left corona radiata hemorrhage (Figures 3a, 3b). Digital subtraction angiography (DSA) yielded high-grade left ICA stenosis with pre-stenotic aneurysmal dilatation, left external carotid artery focal irregularities, bilateral renal artery irregularities, and normal visceral mesenteric arteries (Figure 3c). CT angiogram similarly demonstrated normal visceral mesenteric arteries (Figure 3d).

## Discussion

We have identified three cases radiologically consistent with FMD that span the breadth of clinical presentation, ranging from asymptomatic incidental disease to multifocal disease with significant sequelae such as uncontrolled secondary hypertension, aneurysmal rupture, arterial occlusion, or stroke [9]. Identification of biomarkers that predict significant sequelae could potentially improve clinical decision-making. Large FMD registries and additional studies demonstrate a familial pattern in the condition [1,3,4], implicating potential genetic loci such as *YY1AP1* [10], *PHACTR1* [11], *LRP1* and *ATP2B1* [6], and *COL5A1* [7].

A recent cohort study of 264 individuals [7] has demonstrated potentially clinically significant implications with the *COL5A1* c.1540G>A variant, including associations with dissection, number of affected arteries, and in particular visceral artery dissection and potentially VAA formation commonly identified in the setting of visceral artery disease [12], with VAA 2.7 times higher in FMD patients with the *COL5A1* variant [7].

VAA of the CHA and GDA is rare in the general population [8] and in patients with FMD [13,14], and the natural history of VAA is still poorly defined [15]; a significant proportion present with rupture associated with at least a 10% mortality rate [16]. There are currently no specific monitoring guidelines for subthreshold CHA aneurysms especially in FMD [8,15]. CTA remains the best modality to monitor these lesions, carrying with it cumulative risks associated with contrast and ionizing radiation. Detection of certain high-risk visceral disease FMD genotypes, e.g., *COL5A1*, may discern within this population those who require a more aggressive surveillance strategy. Future studies determining the specific role of *COL5A1* in the natural history of FMD involving the visceral arteries will identify any potential contribution to the clinical evaluation of visceral FMD.

## Conclusions

Optimization of monitoring guidelines in the setting of VAA in FMD is required. Confirmation of the involvement of genotypes such as *COL5A1*, preliminarily implicated in more aggressive, visceral variants of FMD, through ongoing prospective studies may provide important additional information to refine current surveillance strategies. Further research is required to assess the role of genotyping in FMD surveillance programs.
